# ParC, a New Partitioning Protein, Is Necessary for the Active Form of ParA From *Myxococcus* pMF1 Plasmid

**DOI:** 10.3389/fmicb.2020.623699

**Published:** 2021-01-15

**Authors:** Duohong Sheng, Xiaojing Chen, Yajie Li, Jingjing Wang, Li Zhuo, Yuezhong Li

**Affiliations:** State Key Laboratory of Microbial Technology, Institute of Microbial Technology, Shandong University, Qingdao, China

**Keywords:** *Myxococcus*, pMF1, plasmid partitioning, *parC*, ParA function, protein evolution

## Abstract

The ParABS partitioning system, a main driver of DNA segregation in bacteria, employs two proteins, ParA and ParB, for plasmid partition. The pMF1 plasmid from *Myxococcus fulvus* 124B02 has a *par* operon encoding a small acidic protein, ParC, in addition to type I ParA and ParB homologs. Here, we show that expression of *parC* upstream of *parA* (as in the natural case), but not ectopic expression, is essential for the plasmid inheritance in *Myxococcus* cells. Co-expression of *parC* upstream of *parA* was determined to form a soluble ParC–ParA heterodimer at a 1:1 ratio, while individual expression of *parA* or co-expression of *parA* with ectopic *parC* formed insoluble ParA proteins. Purified ParA proteins alone had no ATPase activity and was easily dimerized, while mixing ParA with ParC formed the ParC–ParA heterodimer with the ATPase and polymerization activities. Fusing ParC and ParA also produced soluble proteins and some chimeras restored the ATPase activity and plasmid inheritance. The results highlight that proximal location of *parC* before *parA* is critical to realize the functions of ParA in the partition of *Myxococcus* plasmid pMF1 and shed light on a new mechanism to realize a protein function by two separate proteins.

## Introduction

The Partitioning (Par) system exists widely in bacteria and archaea, participating in the isolation and allocation of chromosomes and plasmids into daughter cells during cell division (Ogura and Hiraga, 1983; Gerdes et al., 1985; Ebersbach and Gerdes, 2005). A Par system typically consists of three components: an NTPase (usually named ParA), a DNA-binding protein ParB, and a *cis*-acting centromere-like sequence *parS*. ParB binds specifically onto the *parS* sequence and assembles into a partitioning complex, on which the ParA proteins are added by binding to the ParB proteins and act as an energy machinery by hydrolyzing NTP to move the ParB–*parS* complex apart. The *parA* and *parB* genes are usually found to form an operon, with the *parS* elements located within or adjacent to the operon (Gerdes et al., 2010; Dobruk-Serkowska et al., 2012; Badrinarayanan et al., 2015).

According to the ParA sequence similarity, bacterial Par systems are divided into three main types; Type I has a deviant Walker-box ATPase, while type II and type III encode actin and tubulin-like NTPases, respectively (Gerdes et al., 2000; Mierzejewska and Jagura-Burdzy, 2012). Type I Par system is found in most bacterial and archaeal chromosomes and plasmids, and is probably the most ubiquitous partitioning system type in nature. In addition to ATPase activity, Type I ParA has exhibited the ability to aggregate (Barlla et al., 2005; Lim et al., 2005; Hwang et al., 2013), although whether this aggregation played a role in plasmid partition has not been proved. Type I Par system can be further divided into two subfamilies, Ia and Ib. Type Ia ParA, such as the ParA proteins encoded in P1 and F plasmids, contains a Walker-box region and an N-terminal winged helix–turn–helix (HTH) domain, which plays an auto-regulation role for the transcription of *parAB* operon (Gerdes et al., 2000; Dunham et al., 2009). Type Ib ParA contains only the Walker-box domain, represented as the ParA proteins in pTAR, pTP228, pB171, or pSM19035, and their auto-regulation role for Par transcription is fulfilled by the ParB proteins (Kwong et al., 2001; Mierzejewska and Jagura-Burdzy, 2012). Both Ia and Ib ParA proteins use the Walker-box domain to engage in the nucleoid to track for their partitioning functions (Vecchiarelli et al., 2010).

The pMF1 plasmid was discovered in *Myxococcus fulvus* 124B02 and is the first and as yet the sole endogenous plasmid that is able to replicate autonomously in cells of myxobacteria (*Myxococcales*) (Zhao et al., 2008b). The plasmid is 18,634 bp in size, containing 23 genes (*pMF1.1-pMF1.23*). Thirteen of the plasmid genes have their homologs in different sequenced myxobacterial genomes, while the others have not yet found their homologs in the GenBank database (Supplementary Table 1), which suggest that pMF1 has a long-standing co-adaption within myxobacteria (Chen et al., 2016, 2020). We previously determined that the plasmid replication is controlled by the *pMF1.13-pMF1.15* fragment, based on which, shuttle vectors between *Escherichia coli* and *M. xanthus* DZ1 have been successfully constructed (Zhao et al., 2008a,b; Feng et al., 2012). pMF1 employed at least two strategies for its stable inheritance in *Myxococcus*; one is the partitioning system encoded by the *pMF1.21-pMF1.23* genes (Sun et al., 2011), while the second is a post-segregational killing system of nuclease toxin and immune protein encoded by the *pMF1.20* and *pMF1.19* gene pair (Li et al., 2018), which belongs to a large family of myxobacterial specific toxins–immunity proteins called SitA6/SitI6 (Vassallo and Wall, 2019). In the partitioning system, *pMF1.22* is predicted to be a *parA* gene, while *pMF1.23* encodes a DNA-binding protein (*parB*), which is able to specifically bind to the *parS* (Sun et al., 2011). Based on the ParB auto-regulation activity on the expression of the *par* genes and the ParA protein sequence similarity, the pMF1 partitioning system was suggested to be a member of the Ib type (Sun et al., 2011). Specifically, the pMF1 *par* operon has a third function–unknown gene, *pMF1.21* (named *parC*), upstream of *parA* and *parB*. The three genes are in the same transcriptional unit, and *parC* is able to enhance the DNA-binding activity of ParB and negatively regulate gene expression of the *par* locus (Sun et al., 2011; Chen et al., 2016; Li et al., 2018).

In this study, we revealed that the *parC* gene is functionally essential for the partitioning system in *Myxococcus* cells. We determined that *parC* located in front of *parA* is critical for the plasmid inheritance in *Myxococcus* cells. Co-expression of *parC* upstream of *parA* produced a soluble ParC–ParA complex with the typical ParA activities and prevented the formation of insoluble and inactive ParA proteins. Our results highlight that the proximal location of *parC* upstream of *parA* is critical to realize the functions of ParA in the partition of *Myxococcus* plasmid pMF1 and suggest a new protein evolution approach to realize a protein function by two separate proteins.

## Results and Discussion

### *parC* and Its Location in pMF1 *par* Operon Are Critical for the Plasmid Partition

**FIGURE 1:**
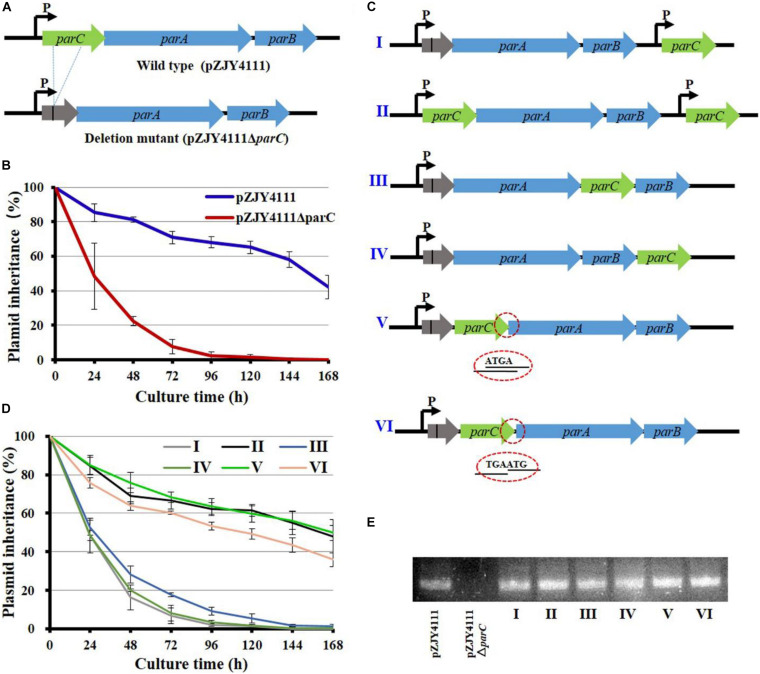
Mutation and organization of the *parC* gene in the shuttle plasmid pZJY4111 and its effects on the inheritance stability of plasmid. **(A)** In-frame deletion of *parC* of the *parCAB* operon in pZJY4111. The deletion removed 228 bases and retained 11 N-terminal bases and 25 C-terminal bases of *parC* (referred to Supplementary Figure 3), producing plasmid pZJY4111Δ*parC*, which was further electro-transformed into *M. xanthus* DZ1 to assay the plasmid retention ability. Arrows indicate the direction of transcription. **(B)** Inheritance stability of pZJY4111 and pZJY4111Δ*parC* in *M. xanthus* DZ1. **(C)** Schematic representation for different locations of the compensatory *parC* gene in pZJY4111Δ*parC* or pZJY4111. These plasmids were separately transformed into *M. xanthus* DZ1 to assay their inheritance stabilities in *M. xanthus* DZ1 **(D)**. Error bars in **(B,D)** indicate the standard deviation (SD) of at least five independent experiments. The DZ1 strains with different plasmids were cultivated in CTT medium without antibiotics for 168 h, and the plasmid maintenance was examined every 24 h. **(E)** Transcriptional confirmation of the expression of *parC* in *M. xanthus* DZ1 containing different plasmids using quantitative RT-PCR.

The *parC*, *parA*, and *parB* genes are organized in turn to form the *parCAB* operon (*pMF1.21-pMF1.23*; gene locations refer to Supplementary Figure 1A). Quantitative PCR amplification indicated that, in the pMF1-harboring *M. fulvus* 124B02 strain, as well as the Par system-containing shuttle plasmid pZJY4111, *parC* transcribed slightly higher than *parA*, but lower than *parB*, which were comparatively at a similar transcriptional level to the replication genes (Supplementary Figure 1B). To determine whether *parC* is required for the partitioning function, we made an in-frame deletion of the *parC* gene in pZJY4111 (Figure 1A), which contains the *ori* region and *par* locus of pMF1. Compared to approximately 60% retention of pZJY4111 in DZ1 strain after 144 h of incubation in the absence of antibiotic selection, the retention of pZJY4111Δ*parC* was dramatically declined to 20% after 48 h, and to almost zero after 96 h of incubation (Figure 1B). The above results indicated that the *parC* gene plays a crucial role in the partitioning system for the plasmid maintenance in *Myxococcus* cells.

We experimentally compensated the deletion by ectopically inserting the *parC* gene with its own promoter in pZJY4111*ΔparC*, producing pZJY4111Δ*parC*:*parC* (plasmid I in Figure 1C). We performed the same insertion of the *parC* gene with its own promoter in the pZJY4111 plasmid, producing pZJY4111:*parC* (plasmid II). There was a 203-bp interval space sequence between the deficient *par* operon and the *parC* compensating gene. Surprisingly, the compensation in plasmid I did not restore the plasmid stability phenotype, while plasmid II exhibited similar inheritance ability to pZJY4111 in *M. xanthus* DZ1 (Figure 1D). To verify potential location effects of *parC* on plasmid stability, we placed *parC* at different places in the *parC*-deficient operon of pZJY4111Δ*parC*, either after *parA*, forming the *parA–parC–parB* cascade (plasmid III); after *parB*, forming the *parA–parB–parC* cascade (plasmid IV); or in front of *parA* and behind the *parC* residuals (plasmid V) (Figure 1C). The plasmid stability assays indicated that plasmid III or IV did not restore the plasmid maintenance, whereas plasmid V completely restored the plasmid stability with almost the same curve as the wild-type plasmid pZJY4111 (Figure 1D).

In the *parCAB* operon, the coding sequences of *parC* and *parA* overlap four bases (ATGA; referred to Supplementary Figure 3), and the inserted *parC* gene in plasmid V also overlapped the four bases with the *parA* gene. To investigate whether the overlap had an effect on partitioning, we added the *parC* gene in front of *parA* by replacing ATGA with TGAATG (plasmid VI; Figure 1C). Plasmid VI reduced the inheritance to some extent as compared with pZJY4111 or *in situ* compensating plasmid V, but the inheritance was insignificantly higher than those plasmids with ectopically inserted *parC* (Figure 1D). The transcriptions of *parC* in *M. xanthus* DZ1 were confirmed by quantitative PCR amplification (Figure 1E). The above results indicated that ectopically expressed or excessive ParC has no effect on the plasmid stability. The function of ParC for the plasmid partitioning strictly depends on the adjacent location of *parC* upstream of *parA*. The co-expression schedule of *parC* and *parA* was probably for interaction of these two proteins.

### *parC* in Front of *parA* Promotes Soluble Expression of ParA by Forming the ParC–ParA Heterodimer

**FIGURE 2:**
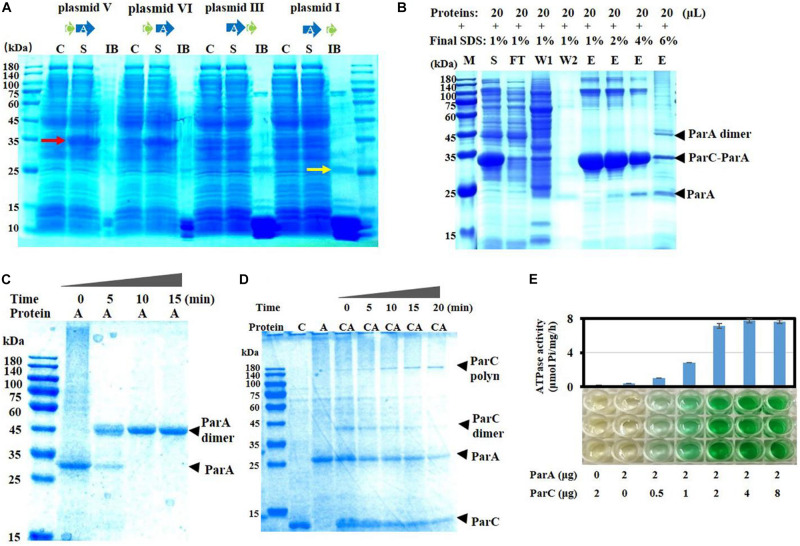
Expression and activities of ParC and ParA proteins. **(A)** The two genes were linked into the expression plasmid pET-15b according to the arrangement sketched in Figure 1C. The recombinant plasmids were transformed into *E. coli* BL21(DE3) and expressed by IPTG induction. After harvesting, cells were washed, lysed, and centrifuged. The supernatant (S) and inclusion body (IB) were analyzed by SDS-PAGE. Non-induced cultures were used as control (C). To aid protein dissolution for electrophoresis, IB was homogenized using a tissue grinder and treated with 6 M guanidine hydrochloride (GuCl). The induced soluble proteins (35 kDa) were pointed by a red arrow, while the insoluble proteins with the ParA size was pointed by a yellow arrow. **(B)** Co-expression of the *parC* (fused with a His-tag) and *parA* genes in *E. coli*. The *parC* and *parA* genes were constructed in pET15b, which was electrotransformed into *E. coli* cells for expression. The expressed His-tagged ParC proteins were purified using Ni-NTA agarose according to the standard protocol (Qiagen, Valencia, CA, United States). The purified proteins were treated with gradient SDS concentrations and analyzed by SDS-PAGE. M, marker; C, control; S, supernatant; IB, inclusion body; FT, flow though; W, washing; E, elution. **(C)** ParA aggregation analysis. ParA monomer was incubated at 32°C for the indicated times (0, 5, 10, and 15 min), and the aggregation was mixed with 0.1% SDS and detected by native-page electrophoresis. **(D)** Interactions between ParA and ParC in binding buffer containing 3 mM ATP, incubated at 32°C. The mixture was sampled at intervals of 0, 5, 10, 15, and 20 min, and detected by electrophoresis. **(E)** ATPase activity assay with ParC and ParA proteins. ATPase activity was measured by detecting the release of Pi at 32°C. Data shown are averages of three repeats. Error bars refer to the SD.

To investigate interactions between ParC and ParA proteins after expression, we constructed their genes into the pET15b expression vector in the same arrangement as they were in the complementing plasmids and expressed them in *E. coli*, respectively. When *parC* and *parA* overlapped four bases (as in plasmid V), an obvious soluble band with approximately 35 kDa in size was induced (pointed by a red arrow in Figure 2A). For the adjacent *parC* and *parA* genes without overlapped bases (as in plasmid VI), the same soluble protein band was observed, but with a small amount of insoluble proteins. However, when *parC* was placed behind *parA* (as in plasmid III) or the two genes were transcribed separately (as in plasmid I), more insoluble proteins appeared (including a 24-kDa band pointed by a yellow arrow in Figure 2A), but no obviously soluble 35-kDa band.

The 35-kDa band was cut, digested with trypsin, and identified by mass spectrometry. The mass–charge ratio (*m*/*z*) data of peptide segments were retrieved against *M. fulvus* 124b02 database, and they were identified as two proteins, ParA (pMF1.22) and ParC (pMF1.21) (Supplementary Figure 3A). The relative abundance of the peak area of peptide segments were quantified and the sum of all the peak area of ParA or ParC was calculated. The coverage ratios of the identified peptide segments were 63 and 63.22% for ParA and ParC. The relative abundance of total peak area of ParA was 2.686 times that of ParC (Supplementary Figure 3B), approximately equaling the amino acid ratio of the two proteins (2.609). Thus, the molar ratio of ParC and ParA in the 35-kDa band is a one-to-one heterodimer and the exact molecular weight of this band should be 32.97 kDa.

We further fused a His-tag at the N-terminus of ParC protein and co-expressed *parA* and the *parC* gene in *E. coli* cells to separate the ParC–ParA complex. The induced ParC and ParA proteins appeared in the supernatant fraction as a soluble heterodimer complex under the 1% SDS condition. After purification with Ni-NTA agarose, there were several bands with high molecular weights (Figure 2B). With the concentration increase of SDS, the ParC–ParA heterodimer, as well as the high-molecular-weight complexes, disaggregated gradually. When treated with 6% SDS, the ParC–ParA heterodimer and the high-molecular-weight complexes were mostly disappeared, accompanying by the appearance of ParA monomer and dimer bands. The results suggested that ParC has the capacity of binding to ParA to form the ParC–ParA heterodimer, and the ParC–ParA heterodimer might be the reason why *parC* and its location in pMF1 *par* operon are critical for the plasmid partition.

### The ParC–ParA Heterodimer Showed the Aggregation and ATPase Activity, but Not ParA Alone

To investigate the potential interaction patterns between ParC and ParA, we constructed their structure models using the i-tasser (Iterative thread assembly optimization) method (Schumacher et al., 2015), and predicted protein–protein docking with the PRISM web server (Pasek et al., 2006). The *Myxococcus* ParA protein was structurally composed of five beta folds in the center and outside around with eight alpha helixes (Supplementary Figure 4A), while ParC consisted of three alpha helixes (Supplementary Figure 4B). There are three potential surface regions for the formation of ParA homodimer with binding energies of −84.24, −36.3, and −33.84 kJ/mol, respectively (labeled I, II, and III in Supplementary Figure 4C). Region III is similar to the dimer binding interface of ParF or Soj, which is involved in the formation of P-Loop, forming an active nucleotide “sandwich” dimer with ATP in the middle (Surtees and Funnell, 2001; Leonard et al., 2005; Schumacher et al., 2012). Region II is on the outside of H7/8, partly overlapped with the binding interface 2 of ParF. Region I locates in the H5–β5–H6 region and has the highest binding energy, leading to the preference of binding upon region I to form ParA homodimer [ParA-(I)-ParA]. Further docking of the ParA homodimer showed that homodimerization on region I reduces the further formation of P-Loop, and the binding energy of Region III decreased to −9.13 kJ/mol (Supplementary Figure 4D).

Two regions on the surface of ParA were predicted for ParC binding, with −36.34 kJ/mol and −14.91 kJ/mol binding energy, respectively (labeled C1 and C2 in Supplementary Figure 4C). C1 is the main binding site of monomers ParA and ParC, locates in the N-terminal region, and covers the region of H5, H5–β5 loop, H6–β6 loop, and C-terminus of H11, which partially overlaps with region I. Binding of ParA with ParC significantly decreases the self-dimerization binding energy of regions I and II, from −84.24 and −36.3 kJ/mol to −12.47 and −12.68 kJ/mol, respectively (Supplementary Figure 4E). Thus, binding with ParC contributes to the further active dimerization at region III (Supplementary Figure 4F).

Next, we expressed ParC and ParA proteins to verify the effect of their binding on the activity of ParA. ParC alone was solubly expressed easily (Supplementary Figure 5A), while it was hard to obtain the soluble form for ParA alone. After many attempts (Supplementary Figure 5B), soluble expression of ParA in *E. coli* was obtained with the MBP fusion expression system, in which a maltose-binding protein was added at the N-terminal of ParA (Supplementary Figure 5C). We removed the MBP tag from the MBP–ParA fusion protein by the proteinase Factor Xa digestion (Supplementary Figure 5C), and the prepared ParA proteins were mostly in monomer form, and turned into dimer with almost no monomer within 10 min at 32°C (Figure 2C). In the dimer, two ParA firmly bound together and SDS treatment did not dissolve the ParA dimer even treated with 6% SDS, 10 mM DTT, or even 6 M guanidine hydrochloride (Supplementary Figure 6). Because monomeric ParA proteins were easily dimerized, in the following *in vitro* experiments, ParA monomer from the MBP–ParA fusion protein was used immediately after digestion, separation, and purification.

It has been reported that type I ParAs have also the ability to form polymer (Kwong et al., 2001; Barlla et al., 2005; Dunham et al., 2009). In order to detect whether the ParC–ParA heterodimer retains ParA activity, the aggregation ability of proteins was tested here. Results showed that ParC or ParA alone could not form an obvious polymer band, while the ParC–ParA heterodimer could form a clear polymer (Figure 2D). With the increase of incubation time at 32°C (0–20 min), the ParC–ParA heterodimer appeared first and then gradually decreased, and a high-molecular-weight band (polymer), which was identified by mass spectrometry as the polymer of ParC and ParA with a ratio of 1:1, appeared and increased (Figure 2D). The ParC–ParA heterodimer migrated to the position of 40 kDa, and the polymer appeared in the range of 140–180 kDa. Therefore, the size of the polymer should be 4 × ParC–ParA heterodimer. Further ATPase experiments found that ParA proteins alone had almost no ATPase activity, but retrieved ATPase activity when mixed with ParC (Figure 2E). The ATPase activity reached the highest at the ParC–ParA ratio of 1:1, and more ParC had no increase to the activity. Thus, ParC and ParA proteins are able to form a heterodimer, which is further polymerized into the ParC–ParA polymer. Co-expressed ParC and ParA proteins in proper organization were able to combine immediately to form a soluble and tightly bound ParC–ParA heterodimer complex, thus preventing the formation of indissoluble ParA homodimer.

### Fusing ParC and ParA Restores Weak Activities of the Typical ParA Proteins

The above results also indicated that the combination of ParC and ParA heterodimer played the function of typical ParA proteins, and ParC bound to the N-terminal (C1 site) of ParA, which may indicate whether they evolved from a protein. The ParC secondary structure is considerably similar to that of the N-terminal fragment of Ia-type ParA (ParA from plasmid P1) (Surtees and Funnell, 2001; Dunham et al., 2009) or the C-terminal fragment of *Sulfolobus* ParB (Schumacher et al., 2015) by containing a predicted Helix–Turn–Helix motif (Supplementary Figure 7). In the structural model of the fusion protein ParCA, the ParC fragment was near the C1 binding site (Supplementary Figure 8). Region I is affected, forming a new N-terminal region for self-dimerization binding (−12.47 kJ/mol). Similar to that in ParA, the ParCA fusion protein retains the II and III self-aggregation regions, but with significantly decreased binding energy of region II (−12.68 kJ/mol). It was speculated that the ParCA protein preferentially forms homodimer at site III and thus has ATPase activity.

**FIGURE 3:**
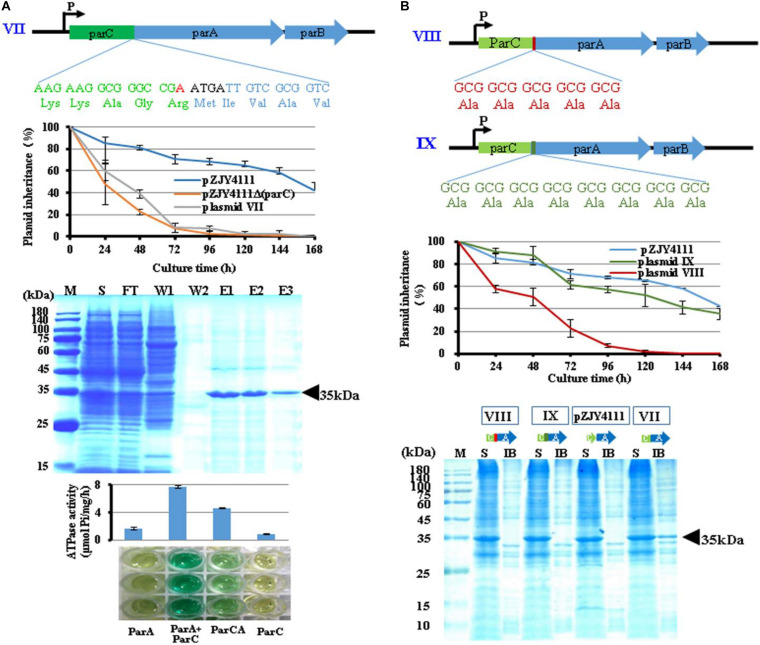
Properties of ParCA fusion proteins. **(A)** An adenylate (red) was inserted before the overlapping region (ATGA) to destruct the stop codon (TGA) of *parC*, forming a fused ParCA (plasmid VII). The ParCA fusion protein was expressed in *E. coli* BL21(DE3) cells after IPTG induction and further purified. **(B)** Adding a 5 × Ala linker (VIII) or an 8 × Ala linker (IX) between domains C and A in ParCA fusion protein. Inheritance stabilities of different plasmids were assayed in *M. xanthus* DZ1. Error bars indicate the SD of four or six independent experiments. Soluble expression of the ParCA fusion proteins with a 5 × Ala linker (VIII) or an 8 × Ala linker (IX) between the C and A domains. M, protein standard; S, supernatant; IB, inclusion body; FT, flow through; W, washing with 20 mM imidazole; E, elution.

To test whether fused ParCA proteins also possessed the function, we fused the ParC and ParA proteins by adding an A base before the overlapping area (ATGA) of *parC* and *parA* to destroy the stop codon of *parC* (TGA) (plasmid VII) (Figure 3A). Compared to the wild-type pZJY4111 plasmid, plasmid VII had a much low retention capacity in *M. xanthus* DZ1, which was at a similar level to the *parC* deletion mutant. The ParCA fusion protein was partially soluble in *E. coli*, together with some inclusion bodies. Interestingly, ParCA exhibited ATPase activity, which, however, was much lower than that of the ParC–ParA heterodimer. The results suggested that ParC did increase the solubility of ParA proteins, but probably due to the inflexibility between the ParC and ParA fragments, the fused ParCA proteins exhibited weak activities.

To improve the flexibility between the ParC and ParA fragments in the fusion protein, we added a five-alanine or eight-alanine hinge between them (VIII and IX in Figure 3B). The two fusing proteins were almost completely soluble in *E. coli*. While plasmid VIII had no significant effect on plasmid stability, the plasmid IX with a longer amino acid linker improved the plasmid stability in a similar level to pZJY4111.

### The Alkaline Amino Acids in the C-Terminal of ParA Might Be Involved in Its Soluble Expression and Interaction With ParC

**FIGURE 4:**
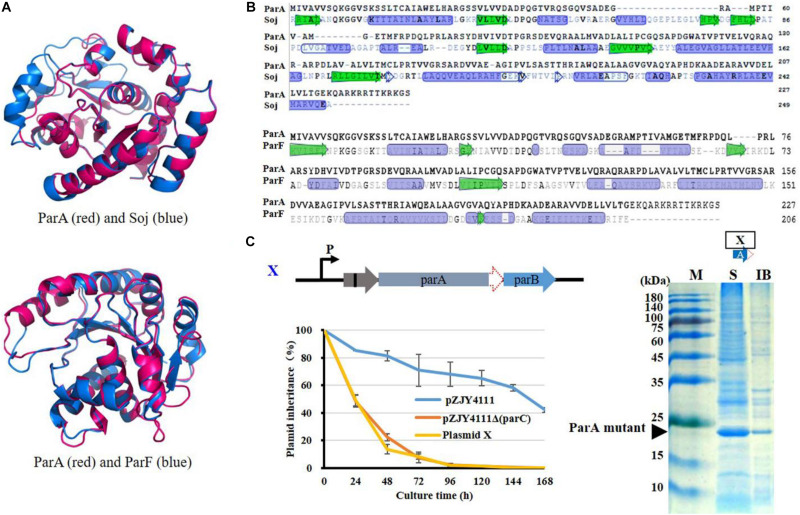
Analysis on structural and functional characteristics of ParA. **(A)** Structural and **(B)** sequence comparison of ParA with Soj of *T. thermophilus* (Leonard et al., 2005) or ParF of *S. newport* TP228 plasmid (Schumacher et al., 2012). Protein structure was predicted with I-TASSER (https://zhanglab.ccmb.med.umich. edu/I-TASSER) (Yang et al., 2015). In the structure comparison, ParA is shown in red and Soj or ParF is shown in blue. **(C)** Deletion of 42 C-terminal bases (14 amino acids) of *parA* of the *parCAB* operon in pZJY4111Δ*parC* (plasmid XI). Inheritance stability of plasmid XI was assayed in *M. xanthus* DZ1. Error bars indicate the SD of four or six independent experiments. Expression of the ParCA fusion proteins was performed in *E. coli* BL21(DE3) cells by using pET15b. ATPase activity was assayed of the ParCA fusion protein. M, protein standard; S, supernatant; IB, inclusion body.

pMF1 ParA has the typical structure of the Walker ATPase protein without the N-terminal HTH domain and belongs to the ParA Ib family, similar to Soj of *Thermus thermophilus* and ParF of *Salmonella newport* TP228 plasmid (Leonard et al., 2005; Schumacher, 2012; Schumacher et al., 2012; Figure 4A). However, compared with typical Ib ParA proteins (Leonard et al., 2005; Schumacher et al., 2012), ParA alone cannot fold correctly and exhibits no ATP activity. Notably, in typical Par systems, ParA is usually an acidic protein. However, in the pMF1 Par system, ParA is also an alkaline protein (the theoretical pI is 8.90, calculated with the ExPASy-Compute pI/Mw tool), which is mainly attributed by the rich basic amino acids in the C-terminal (Figure 4B). Interestingly, ParC is an acidic protein (pI is 4.57), and structurally, its primary binding site C1 covers the C-terminal of ParA (Supplementary Figure 4). The theoretical pI of the ParC–ParA heterodimer is 5.68.

To determine whether the C-terminal sequence was critical for ParA’s expression and functions, we prepared the C-terminal deletion mutant of ParA by removing 14 C-terminal amino acids. Without the alkaline C-terminal tail, the ParA mutant proteins were solubly expressed in *E. coli*, suggesting that the C-terminal basic tail of ParA disturbed its soluble expression. In the pZJY4111Δ*parC* plasmid, the deletion of the alkaline C-terminal of ParA had no contribution for the plasmid stability in *Myxococcus* cells (Figure 4C). Thus, the ParA mutant alone was soluble, but has no partitioning function, which suggested that the combination of ParC to ParA was necessary for the expression and functions of ParA.

ParC, which has no significant homology with any known proteins, consists of three helixes in its secondary structure, and approximately 80% amino acids in the protein participate in the formation of the alpha helix (Supplementary Figure 4B). In the polycistronic mRNA of *parCAB* of pMF1plasmid, ParC is translated first and then binds the nascent ParA polypeptide at a 1:1 ratio as it emerges from the ribosome, which prevents the inactive ParA homodimerization, and participates in the process of plasmid partitioning. Structurally, ParC showed a HTH motif and its primary binding region (C1 region) covers the C- and N-terminal of ParA. The fusion of ParC at the N-terminal of ParA restored the expression and function of ParA. Notably, ParC was also reported to be involved in Par regulation (Sun et al., 2011), which is usually the function of the N-terminus of Ia-type ParA. Further, the ParCA fusion protein sequences were used for searching homologous proteins by the BLASTp program in the GenBank, and a 318-aa putative Ia ParA (Bpet4565) from *Bordetella petrii* was found to be similar to ParCA (Supplementary Figure 8). Thus, ParC unlocks activities that ParA deserves and seems to be an N-terminus subunit of a complete Ia-type ParA protein for expression and function.

Gene fusion/fission is a major contributor to the evolution of multi-domain proteins (Pasek et al., 2006). Here, ParC and ParA function as a whole, but separate into two proteins, which probably provides more interaction plasticity and thus is more efficient than one fusion protein in plasmid partitioning. Their interfaces can thus be changed in a larger range, which contributes to better protein–protein interactions and increases the protein activity and precision adjustability. This is a novel regulation method of the bacterial Par system, different from any known Ia or Ib ParA proteins and will be a good model for studying the function of type I ParA.

## Materials and Methods

### Strains, Plasmids, and Culture Conditions

The strains and plasmids used in this study are listed in Supplementary Table 2. The *M. fulvus* 124B02 and its derived strains were cultivated routinely in CTT medium at 30°C (Hodgkin and Kaiser, 1977). The *E. coli* strains were cultivated in LB medium at 37°C. When required, 40 μg/ml of kanamycin and 100 μg/ml ampicillin were added into the medium.

### Plasmid Curing

The principle that the plasmids with the same *ori* region or *par* region are incompatible was employed to cure pMF1 (Novick, 1987; Austin and Nordstrom, 1990). The plasmids of pZJY4111 [also named pXS11, constructed by Sun et al. (2011)] and pMF1 shared the same *ori* and *par* loci. The pMF1 curing in *M. fulvus* 124B02 was conducted using the protocol as previously described with some modifications (Liu et al., 2012). The shuttling plasmid pZJY4111 was introduced into *M. fulvus* 124B02 with pMF1 by electroporation (400 Ω, 25 μF, 1.25 kV). Then, the cells were grown at 30°C for 4 h in CTT broth without antibiotics on a shaker (200 rpm), followed by spreading onto kanamycin-containing (40 μg/ml) CTT agar plates. After 5- to 6-day incubation, resistant clones were re-transferred onto kanamycin-containing agar plates. Then, three clones were selected, scattered by magnetic beads, and incubated in 50 ml of kanamycin-containing CTT broth (30°C, 200 rpm) for 24 h. After transferring seven times, aliquots of each culture were diluted and spread onto kanamycin-containing agar plates. To determine the elimination of pMF1 and the presence of pZJY4111, the total genomic DNA of some clones was extracted and analyzed by PCR amplification of different regions. The primers used in this study are listed in Supplementary Table 3. Positive clones were selected and designated as *M. fulvus* 124B02/pZJY4111.

Next, the 124B02/pZJY4111 was cultured in 50 ml of CTT broth without antibiotics, transferred as above for seven times, and spread on plates without antibiotics. After 6-day incubation, single clones were transferred onto plates with and without kanamycin to screen plasmid-free candidates.

### Construction of *parC* Knockout, Compensation, and Overexpression Plasmids

Based on the pZJY4111 plasmid, the deletion, compensation, and overexpression plasmids of the *parC* gene were constructed by PCR and DNA ligation. The used primers are listed in Supplementary Table 3. These plasmids were introduced into *M. xanthus* DZ1 by electroporation for further plasmid stability assay.

### Plasmid Stability Assay

To test the stability, *M. xanthus* DZ1 strains harboring the plasmids were grown to the late exponential phase in liquid CTT medium supplemented with 40 μg/ml kanamycin. Then, we diluted the cultures by 1:25 in fresh CTT liquid medium with no antibiotics and grown at 30°C and 200 rpm. After 24 h of incubation, the cultures were serially diluted and plated on CTT agar without antibiotics. The dilutions and plating were routinely repeated every 24 h until 168 h of incubation. In each round, 100 single colonies were patched onto CTT agar with and without kanamycin, and the plasmid stability was measured as the percentage of antibiotic-resistant clones (Sun et al., 2011).

### Protein Expression and Purification

The proteins were expressed in *E. coli* BL21 (DE3), induced by the addition of 0.1 mM of IPTG when the OD_600_ value of the culture reached 1.0. The BL21 cells were grown at 37°C in LB broth with antibiotics. After the addition of IPTG, the cultures were grown at 16°C for 20 h. The cells were then collected and resuspended in lysis buffer (25 mM Tris–HCl, pH 8.0, 200 mM NaCl, and 5% glycerol, pH 8.0) and lysed via ultra-sonication. The mixtures were centrifuged at 4°C for 30 min at 12,000 rpm. The soluble proteins were mixed with amylose resin (New England Biolabs) according to the manufacturer’s protocols.

SDS polyacrylamide gel electrophoresis (SDS-PAGE) was used to investigate the purified protein. Protein molecular weight markers (Takara) were used to estimate the molecular weight. Native-PAGE was performed using a polyacrylamide gel without SDS and ran at 150 V with a constant current of 15 mA/gel for 4 h in a 4°C cold room. All proteins separated by PAGE were visualized using Coomassie Brilliant Blue staining, and imaged on a Typhoon^TM^ Variable Mode Imager (GE Healthcare).

### Structural Modeling

I-TASSER was used to model the structure of ParA and ParC (Yang et al., 2015). I-TASSER is a hierarchical method for protein structure prediction, utilizing the most similar structural templates to predict the most reliable models. The best model was chosen according to the C-score, which is calculated based on the threading template alignments and structural assembly simulations. Structural templates were first identified from the PDB by the multiple-threading program LOMETS; then, full-length models were constructed by iterative template fragment assembly simulations. All the structural models were refined in the atomic level by the fragment-guided molecular dynamics (FG-MD) simulations (Zhang et al., 2011). The modeled structure was displayed by PyMOL.

### ATPase Assay

The ATPase activity measurements were performed spectrophotometrically using the ultra-micro total ATPase detection kit (Jiancheng Bio. Nanjing), as previously shown (Sheng et al., 2005).

### Polymerization Assay

ParA polymerization assay was performed as previously described (Leonard et al., 2005) with some modifications. ParA protein (6 μg) was incubated in polymerization buffer (50 mM Tris–HCl, pH 7.0, 100 mM KCl, 5 mM MgCl_2_, 2 mM DTT, 10% glycerol, and 1 mM ATP) for 15 min at 30°C. In ParC co-aggregation assays, Gradient ParC was added to the reacting solution at the concentrations indicated. After the reaction was completed, the samples were analyzed by native-PAGE and Coomassie blue staining.

## Data Availability Statement

The datasets presented in this study can be found in online repositories. The names of the repository/repositories and accession number(s) can be found in the article/Supplementary Material.

## Author Contributions

DS and YuL designed the experiments. DS, XC, YaL, JW, and LZ performed the experiments. DS, XC, and YuL analyzed the data. DS and YuL wrote the manuscript. All authors contributed to the article and approved the submitted version.

## Conflict of Interest

The authors declare that the research was conducted in the absence of any commercial or financial relationships that could be construed as a potential conflict of interest.

## Supplementary Material

Supplementary Figure 1 Genes in plasmid pMF1. **(A)** Organization of the genes in plasmid pMF1 and their homologous relationships with genes in myxobacterial genomes. Pink arrows (→), Seven hypothetical genes, each having a single homolog in *M. stipitatus* DSM 14675. Green arrows (→), Three genes, each having many homologs in different genomes only of myxobacteria. Blue arrows (→), Three genes, each having a single homolog in many different genomes, but the genes with the highest similarity come from myxobacteria. Gray arrows (→), Ten genes, having no homology in the survey. Details are referred to Supplementary Table 1. The genes of *repA*, *repB*, *repC* (*pMF1.13*-*pMF1.15*), *parC*, *parA*, *parB* (*pMF1.21*-*pMF1.23*), *toxP*, and *immP* (*pMF1.20* and *pMF1.19*) have been determined to involve in the plasmid replication, the plasmid partition, and a post-segregational killing system for plasmid stable inheritance (Zhao et al., 2008a; Sun et al., 2011; Li et al., 2018). **(B)** Quantitative transcription analysis of the partitioning genes using quantitative RT-PCR. The replication genes were inspected as a control and primers used for intergenic region are listed in Supplementary Table 4.

Supplementary Figure 2 The *parC* gene and the corresponding ParC protein sequences, showing the sites for the in-frame deletion. The missing part was marked in red, the starting codon and amino acid were framed in green, the ending codon was framed in purple, and the ribosome binding sites was underlined in green.

Supplementary Figure 3 Identification of the induced 35 kDa protein by mass spectrometry. The induced protein bands (pointed by a red arrow in Figure 2A) were cut, digested by trypsin and identified by mass spectrometry. **(A)** Peptide peaks and their mass-charge spectra are shown. **(B)** The list of identified peptides.

Supplementary Figure 4 Structural basis for the protein interaction of ParA and ParC, and effects of the binding of ParC on the ParA dimerization. **(A)** Structure of ParA, predicted with I-TASSER (https://zhanglab.ccmb.med.umich.edu/I-TASSER). *Myxococcal* ParA structure contains β1(residues 1–6) – H1(residues14-27) – β2(residues31-37) – H2(residues42-46) – H5(residues70-71,75-77) – β5(residues82-87) – H6(residues93-101) – β6(residues103-109) – H7(residues114-115,119-128) – β7(residues137-143) – H8(residues151-161) – H10(residues176-183,187-190) – H11(residues195-213). The arrangement order of sheets and helices follows Soj. **(B)** Structure of ParC. **(C)** Predicted protein-protein interaction sites on the surface of ParA. Three ParA self-dimerization regions (I, II, III) and two ParC-binding regions (C1 and C2) are marked with brown and pink rings, respectively. The binding energy was calculated by PRISM webserver. **(D)** Predicted protein-protein interaction sites of ParA dimer in I region (ParA-(I)-ParA). **(E)** Predicted protein-protein interaction sites on the surface of the ParC-(C1)-ParA heterodimer. **(F)** Docking of two ParC-(C1)-ParA heterodimer. ParC colored with pink. ParA colored rainbow in the cartoon model and blue and green in the schematic diagram, respectively.

Supplementary Figure 5 Electrophoresis assay of the expressions of ParC and ParA proteins in *E. coli*. **(A)** Expression of *parC* in the pET15b expression system. **(B)** Expression of ParA with different expression vectors, including pET15b, pET30a, pGEX-6p-1, pGro7, pKJE7, pG-Tf2, pG-KJE8, and pTf16. **(C)** Expression of *parA* in the pMAL-c2x expression system. The recombination plasmids were separately transformed into *E. coli* BL21(DE3) and expressed by IPTG induction. Cells were harvested, broken, centrifuged, and the resulting whole cell protein (WCP), supernatant (S) and inclusion body (IB) were analyzed by SDS-PAGE. Non-induced cells were used as control (C).

Supplementary Figure 6 Dissociation analysis of ParA homodimer with gradient SDS concentrations, combined with 10 mM DTT or 6M guanidine hydrochloride (GuCl), which was detected by electrophoresis.

Supplementary Figure 7 Similarity analysis of ParC. **(A)** Structure comparison of ParC, N-terminal of Ia-type ParA, and C-terminal of ParB. Type Ia ParA is from plasmid P1 (PDB ID: 3EZ7.1), and ParB is from *Sulfolobus* pNOB8 (PDB ID: 4RS7). **(B)** Multiple sequence alignment of Ib-type ParF from plasmid TP228, ParA from pMF1 plasmid, ParCA, and Ia-type ParA from P1 plasmid. The N-terminus of ParA protein was marked with red square brackets.

Supplementary Figure 8 Structure model of ParCA fusion protein. **(A)** Structure prediction of ParCA fusion protein with I-TASSER server (Surtees and Funnell, 2001). Three surface self-dimerization sites are marked with red circle and their binding energy were calculated by PRISM protein–protein docking server (Schumacher et al., 2015). **(B)** ParCA docking on the self-dimerization site III.

Supplementary Figure 9 Alignment of ParCA and its homologous protein Bpet4565 from *Bordetella petrii*. Bpet4565 is a putative partition protein homologous to ParCA, found in the GenBank by the BLASTp. Their N-terminal structure is marked in blue box. Alpha helix structures in Bpet4565 (red box) and ParCA (orange box) are marked on their sequence. The identical amino acids are marked by asterisks, and similar amino acids are marked by dots.

Supplementary Table 1 Function annotation of the pMF1 genes in GenBank and the homologous gene sources with the highest similarity.

Supplementary Table 2 Bacterial strains and plasmids used in this study.

Supplementary Table 3 Primers used in this study.
