# Supplementary material for: ParC, a New Partitioning Protein, Is Necessary for the Active Form of ParA From Myxococcus pMF1 Plasmid
Source: Front Microbiol. 2021 Jan 15;11:623699. doi: 10.3389/fmicb.2020.623699 (PMC7843461; doi:10.3389/fmicb.2020.623699)
Supplement: Supplementary Figure 1 — Genes in plasmid pMF1. (A) Organization of the genes in plasmid pMF1 and their homologous relationships with genes in myxobacterial genomes. Pink arrows (→), Seven hypothetical genes, each having a single homolog in M. stipitatus DSM 14675. Green arrows (→), Three genes, each having many homologs in different genomes only of myxobacteria. Blue arrows (→), Three genes, each having a single homolog in many different genomes, but the genes with the highest similarity come from myxobacteria. Gray arrows (→), Ten genes, having no homology in the survey. Details are referred to Supplementary Table 1. The genes of repA, repB, repC (pMF1.13-pMF1.15), parC, parA, parB (pMF1.21-pMF1.23), toxP, and immP (pMF1.20 and pMF1.19) have been determined to involve in the plasmid replication, the plasmid partition, and a post-segregational killing system for plasmid stable inheritance (Zhao et al., 2008a; Sun et al., 2011; Li et al., 2018). (B) Quantitative transcription analysis of the partitioning genes using quantitative RT-PCR. The replication genes were inspected as a control and primers used for intergenic region are listed in Supplementary Table 4. [file Data_Sheet_1.pdf]

Supplementary figures

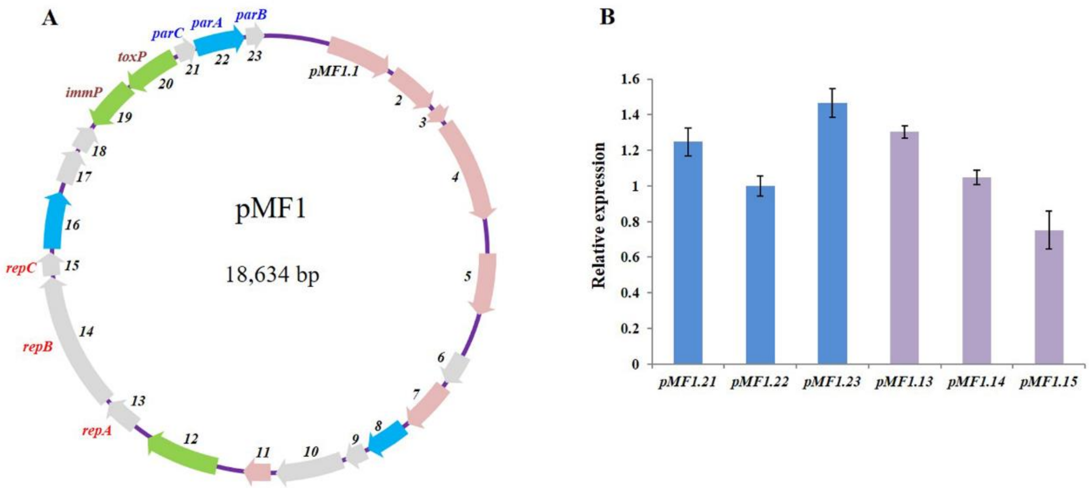

Figure S1. Genes in plasmid pMF1. (**A**) Organization of the genes in plasmid pMF1 and their homologous relationships with genes in myxobacterial genomes. Pink arrows (➡), Seven hypothetical genes, each having a single homolog in *M. stipitatus* DSM 14675. Green arrows (➡), Three genes, each having many homologs in different genomes only of myxobacteria. Blue arrows (➡), Three genes, each having a single homolog in many different genomes, but the genes with the highest similarity come from myxobacteria. Gray arrows (➡), Ten genes, having no homology in the survey. Details are referred to **Table S1**. The genes of *repA*, *repB*, *repC* (*pMF1.13*-*pMF1.15*), *parC*, *parA*, *parB* (*pMF1.21*-*pMF1.23*), *toxP* and *immP* (*pMF1.20* and *pMF1.19*) have been determined to involve in the plasmid replication, the plasmid partition, and a post-segregational killing system for plasmid stable inheritance [19-21]. (**B**) Quantitative transcription analysis of the partitioning genes using quantitative RT-PCR. The replication genes were inspected as a control and primers used for intergenic region are listed in **Table S4**.

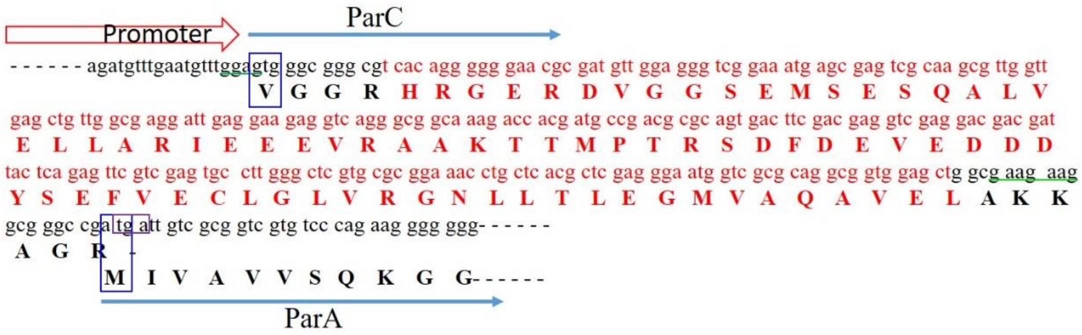

Figure S2. The *parC* gene and the corresponding ParC protein sequences, showing the sites for the in-frame deletion. The missing part was marked in red, the starting codon and amino acid were framed in green, the ending codon was framed in purple, and the ribosome binding sites was underlined in green.

A

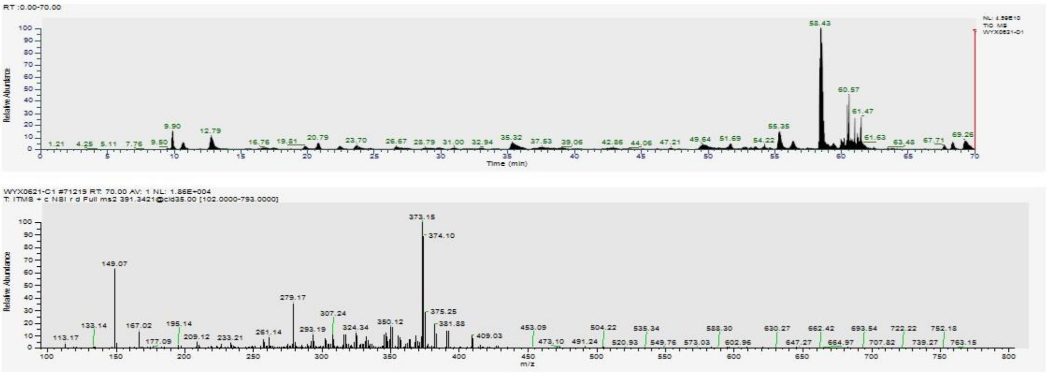

Accession	Description	Score	Coverage	# Proteins	# Unique Peptides	# Peptides	# PSMs	# AAs	MW [kDa]	calc. pI
lcl	[gene=pMF1.22] [protein_id=ABX46805.1]	307.16	63.00	1	15	15	137	227	24.1	8.68
lcl	[gene=pMF1.21] [protein_id=ABX46804.1]	121.79	63.22	1	10	10	36	87	9.6	4.64

B

Proteins	Sequence	# PSMs	RT [min]	Peak Area
ParA (pMF1.22)	IAWQEALAAGVGVAQYAPHDK	2	60.144441	207317559.8088950
	AVVDELLVLTGEKQAR	2	60.045241	154408793.5344020
	DVVAEAGIPVLSASTTHR	17	56.331192	172472927.5088610
	AVVDELLVLTGEK	10	60.572935	1184418006.3034800
	SARDVVAEAGIPVLSASTTHR	5	58.936139	92770768.1921110
	GSSVLVVDADPQGTVR	11	51.654298	141769494.4591480
	IAWQEALAAGVGVAQYAPHDKAADEAR	4	59.903143	234233.8703515
	SYDHVIVDTPGRSDEVQR	37	41.690682	17788993.5548161
	AMPTIVAMGETMFRPDQLPR	3	60.493955	703788455.9781540
	SYDHVIVDTPGR	23	35.989731	141253348.2160020
	MIVAVVSQK	3	37.71741	53418331.6569201
	QSGQVSADEGR	9	9.7212077	23606731.9493446
	mIVAVVSQK	8	35.547045	182508693.9095730
	MIVAVVSQKGGVSK	2	35.138386	33740.0649059
	AMPTIVAmGETMFRPDQLPR	1	59.922992	146732004.9614560
	Total			3222522083.9684200
ParC (pMF1.21)	DVGGSEMSESQALVELLAR	15	62.967268	14334544.9197072
	DVGGSEmSESQALVELLAR	7	60.715289	245590572.7582930
	GERDVGGSEMSESQALVELLAR	3	60.342367	64998590.3042111
	GNLLTLEGMVAQAVELAK	1	61.47329	731923912.6769420
	IEEEVRAAK	2	11.75345	5468852.9225785
	AAKTTMPTR	1	9.0252955	11159.3644900
	GERDVGGSEmSESQALVELLAR	1	60.05201	103094240.5210260
	IEEEVR	4	14.605364	14371398.4660666
	TTMPTR	1	10.330141	6007622.0527220
	TTmPTR	1	11.46638	13746384.2900000
	Total			1199547278.2760400

Figure S3. Identification of the induced 35 kDa protein by mass spectrometry. The induced protein bands (pointed by a red arrow in **Figure 2A**) were cut, digested by trypsin and identified by mass spectrometry. (**A**) Peptide peaks and their mass-charge spectra are shown. (**B**) The list of identified peptides.

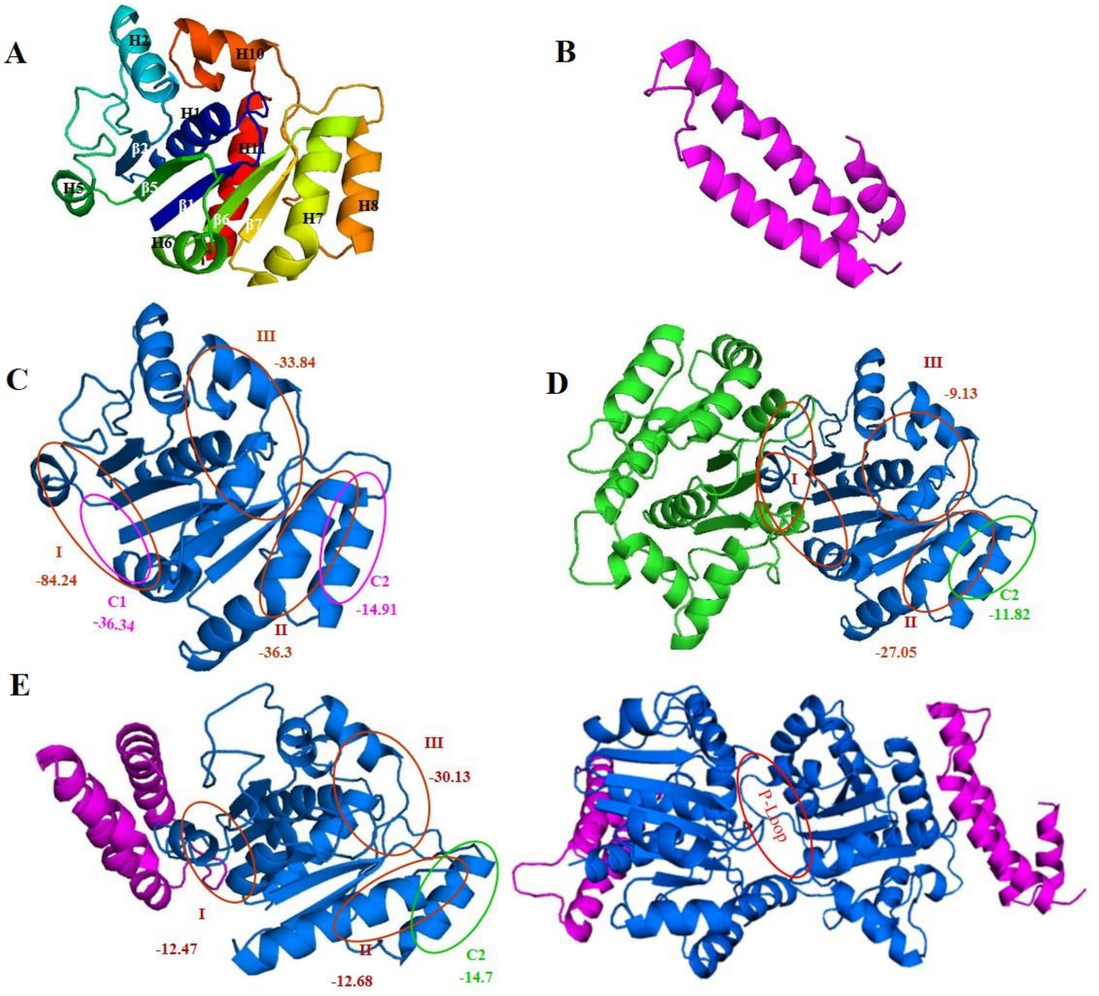

Figure S4. Structural basis for the protein interaction of ParA and ParC, and effects of the binding of ParC on the ParA dimerization. (**A**) Structure of ParA, predicted with I-TASSER (https://zhanglab.ccmb.med.umich.edu/I-TASSER). *Myxococcal* ParA structure contains β1(residues 1–6) - H1(residues14-27) - β2(residues31-37) - H2(residues42-46) – H5(residues70-71,75-77) - β5(residues82-87)- H6(residues93-101) - β6(residues103-109) – H7(residues114-115,119-128) - β7(residues137-143) – H8(residues151-161) – H10(residues176-183,187-190) – H11(residues195-213). The arrangement order of sheets and helices follows Soj. (**B**) Structure of ParC. (C) Predicted protein-protein interaction sites on the surface of ParA. Three ParA self-

dimerization regions (I, II, III) and two ParC-binding regions (C1 and C2) are marked with brown and pink rings, respectively. The binding energy was calculated by PRISM webserver. (**D**) Predicted protein-protein interaction sites of ParA dimer in I region (ParA-(I)-ParA). (**E**) Predicted protein-protein interaction sites on the surface of the ParC-(C1)-ParA heterodimer. (**F**) Docking of two ParC-(C1)-ParA heterodimer. ParC colored with pink. ParA colored rainbow in the cartoon model and blue and green in the schematic diagram respectively.

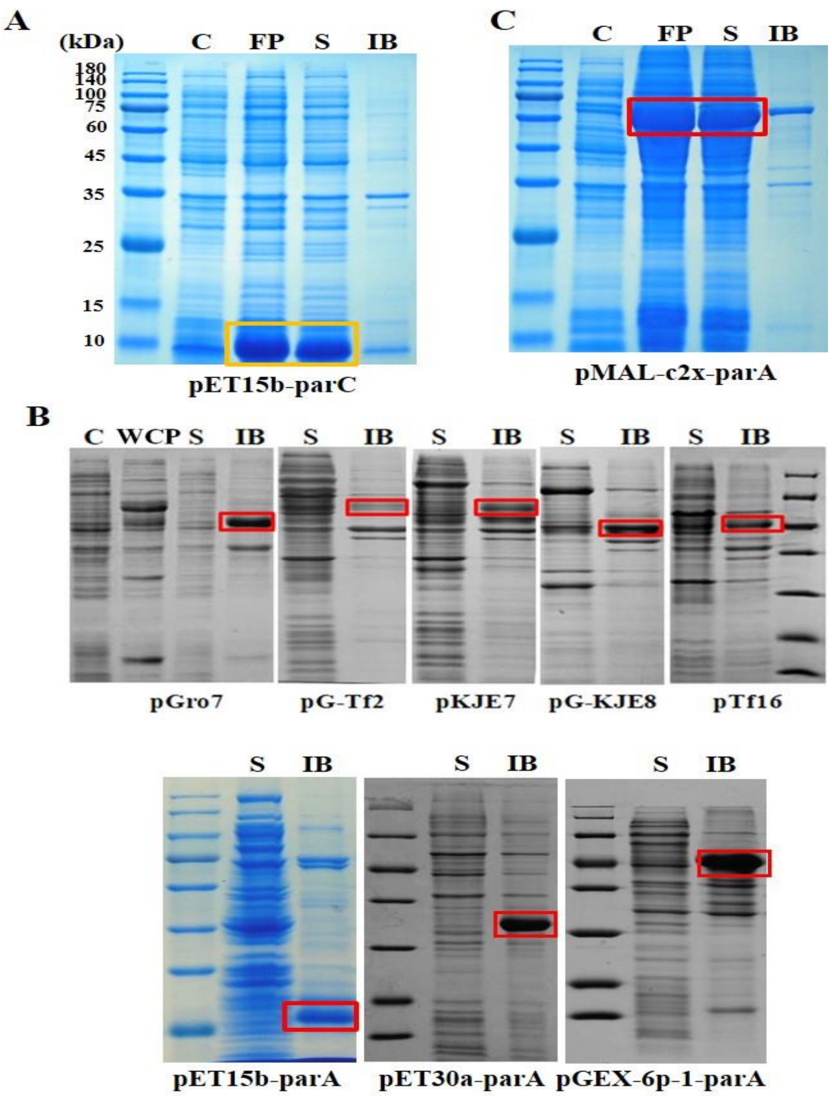

Figure S5. Electrophoresis assay of the expressions of ParC and ParA proteins in *E. coli*. (**A**) Expression of *parC* in the pET15b expression system. (**B**) Expression of ParA with different expression vectors, including pET15b, pET30a, pGEX-6p-1, pGro7, pKJE7, pG-Tf2, pG-KJE8 and pTf16. (**C**) Expression of *parA* in the pMAL-c2x expression system. The recombination plasmids were separately transformed into *E. coli* BL21(DE3) and expressed by IPTG induction. Cells were harvested, broken, centrifuged, and the resulting whole cell protein (WCP), supernatant (S) and inclusion body (IB) were analyzed by SDS-PAGE. Non-induced cells were used as control (C).

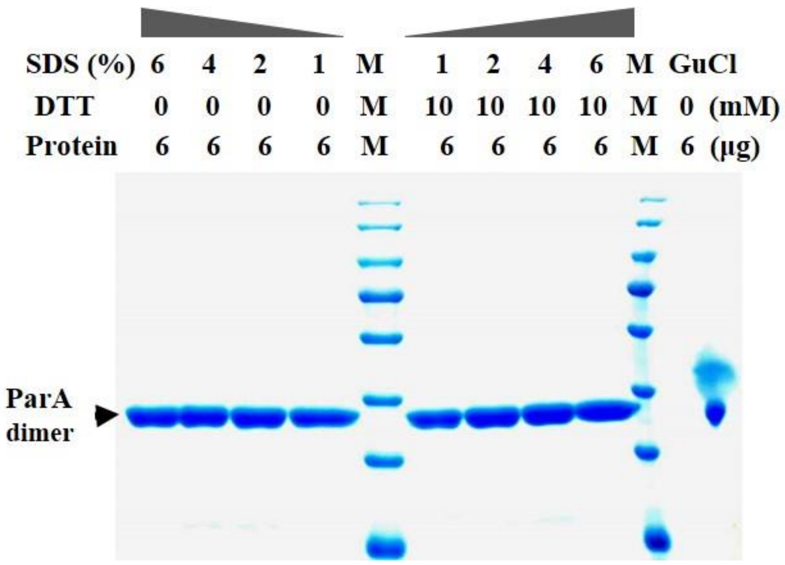

Figure S6. Dissociation analysis of ParA homodimer with gradient SDS concentrations, combined with 10 mM DTT or 6M guanidine hydrochloride (GuCl), which was detected by electrophoresis.

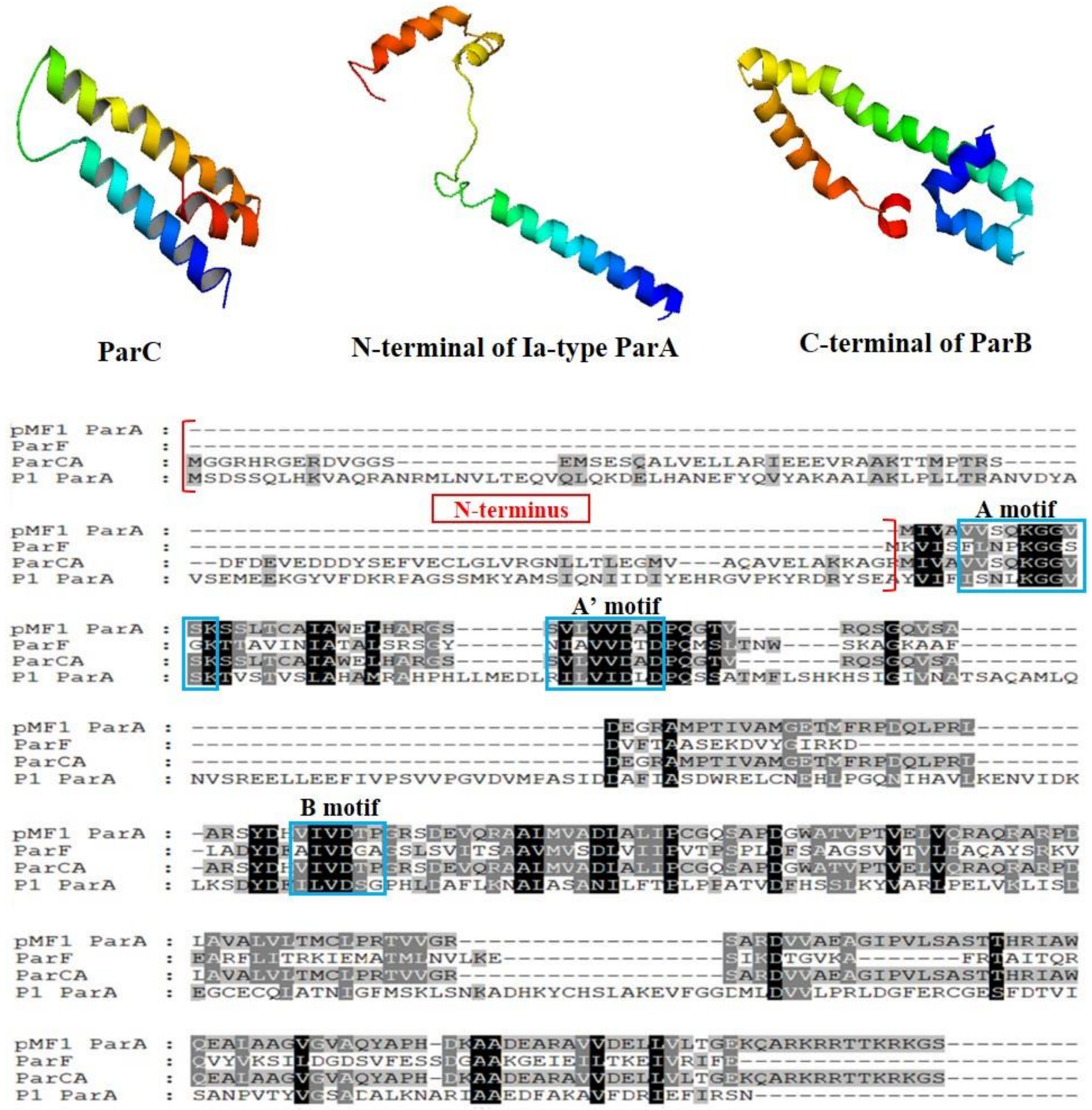

Figure S7. Similarity analysis of ParC. (**A**) Structure comparison of ParC, N-terminal of Ia-type ParA, and C-terminal of ParB. Type Ia ParA is from plasmid P1 (PDB ID: 3EZ7.1), and ParB is from *Sulfolobus* pNOB8 (PDB ID: 4RS7). (**B**) Multiple sequence alignment of Ib-type ParF from plasmid TP228, ParA from pMF1 plasmid, ParCA, and Ia-type ParA from P1 plasmid. The N-terminus of ParA protein was marked with red square brackets.

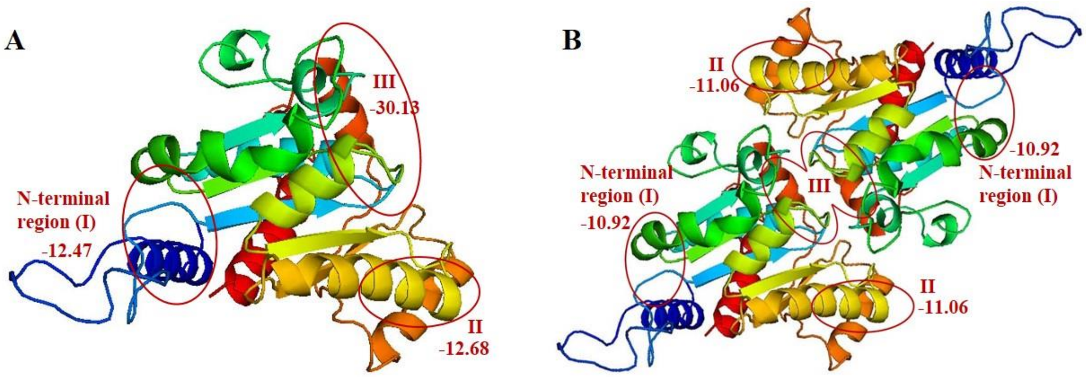

Figure S8. Structure model of ParCA fusion protein. (**A**) Structure prediction of ParCA fusion protein with I-TASSER server [26]. Three surface self-dimerization sites are marked with red circle and their binding energy were calculated by PRISM protein–protein docking server [27]. (**B**) ParCA docking on the self-dimerization site III.

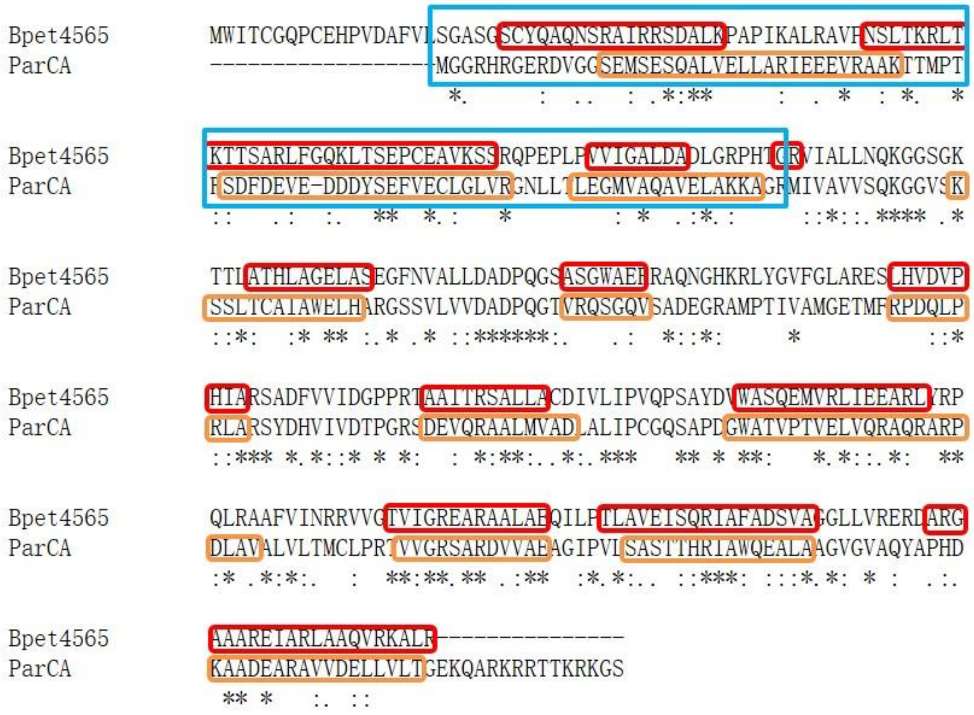

Figure S9. Alignment of ParCA and its homologous protein Bpet4565 from *Bordetella petrii*. Bpet4565 is a putative partition protein homologous to ParCA, found in the GenBank by the BLASTp. Their N-terminal structure is marked in blue box. Alpha helix structures in Bpet4565 (red box) and ParCA (orange box) are marked on their sequence. The identical amino acids are marked by asterisks, and similar amino acids are marked by dots.

Supplementary tables

Table S1. Function annotation of the pMF1 genes in GenBank and the homologous gene sources with the highest similarity.

CDS	Product	Source
pMF1.1	hypothetical protein MYSTI_04154	[*Myxococcus stipitatus* DSM 14675]
pMF1.2	hypothetical protein MYSTI_04155	[*Myxococcus stipitatus* DSM 14675]
pMF1.3	hypothetical protein MYSTI_04156	[*Myxococcus stipitatus* DSM 14675]
pMF1.4	hypothetical protein MYSTI_04157	[*Myxococcus stipitatus* DSM 14675]
pMF1.5	hypothetical protein MYSTI_04158	[*Myxococcus stipitatus* DSM 14675]
pMF1.6		
pMF1.7	hypothetical protein MYSTI_04162	[*Myxococcus stipitatus* DSM 14675]
pMF1.8	Membrane-bound lytic murein transglycosylase D precursor	
pMF1.9		
pMF1.10		
pMF1.11	hypothetical protein MYSTI_04164	[*Myxococcus stipitatus* DSM 14675]
pMF1.12	protein kinase	[*Stigmatella aurantiaca*]
pMF1.13	RepA	
pMF1.14	RepB (replication initiation protein)	
pMF1.15	hypothetical protein MYSTI_04153 RepC	[*Myxococcus stipitatus* DSM 14675]
pMF1.16	resolvase domain-containing protein RepD	[*Anaeromyxobacter* sp. K]
pMF1.17		
pMF1.18		
pMF1.19c	Hypothetical protein MFUL124B02_18095	[*Myxococcus fulvus* 124B02]
pMF1.20c	Hypothetical protein MFUL124B02_18100	[*Myxococcus fulvus* 124B02]
pMF1.21	ParC	
pMF1.22	ParA (partition ATPase)	[*Sorangium cellulosum*]
pMF1.23	ParB (partition DNA binding protein)	

Table S2. Bacterial strains and plasmids used in this study.

Strain or plasmid	Relevant characteristics	Source
**Bacterial strains**		
***E.coli* BL21 (DE3)**	expression strain, λ(DE3 [lacI lacUV5-T7 gene 1 ind1 sam7 nin5]) [malB+]K-12(λS)	Novagen
***E.coli* BL21 (DE3) pLysS**	expression strain, F^- ompT gal dcm lon hsdSB(rB^- mB^-) λ(DE3) pLysS(cm^R)	Novagen
***M.xanthus* DZ1**	non-motile, Non-fruiting, dispersed-growing	Prof. DR. Zusman
***M.fulvus* 124B02**	wild-type, with plasmid pMF1	Stored in the laboratory
***M.fulvus* 124B02/free**	*M. fulvus* 124B02 without pMF1	This study
**Plasmids**		
**pMF1**	a cryptic plasmid from *M. fulvus* 124B02	Zhao et al., 2008
**pZJY4111**	also named pXS11, *E. coli- M. xanthus* shuttle plasmid containing ori and *par* loci of pMF1	Sun et al., 2011
**pZJY4111Δ*parC***	plasmid pZJY4111 with *parC* mutation	Sun et al., 2011
**pG-KJE8**	chaperone expression plasmid, *dnaK-dnaJ–grpE, groES-groEL*	Takara
**pGro7**	chaperone expression plasmid, *groES-groEL*	Takara
**pKJE7**	chaperone expression plasmid, *dnaK-dnaJ–grpE*	Takara
**pG-Tf2**	chaperone expression plasmid, *groES-groEL-tig*	Takara
**pTf16**	chaperone expression plasmid, *tig*	Takara
**pET3a**	*E. coli* expression vector	Novagen
**pET15b**	*E. coli* expression vector	Novagen
**pET29b**	*E. coli* expression vector	Novagen
**pGEX-6p-1**	*E. coli* expression vector	GE
**pMAL-c5x**	*E. coli* expression vector	New England Biolabs
**Plasmid I**	pZJY4111Δ*parC* with a *parC* compensation ex- *par* operon	This study
**Plasmid II**	pZJY4111with a *parC* compensation ex- *par* operon	This study
**Plasmid III**	pZJY4111Δ*parC* with a *parC* compensation after *parA*	This study
**Plasmid IV**	pZJY4111Δ*parC* with a *parC* compensation after *parB*	This study
**Plasmid V**	pZJY4111Δ*parC* with a *parC* compensation before *parA* with gene overlap	This study
**Plasmid VI**	pZJY4111Δ*parC* with a *parC* compensation before *parA* without gene overlap	This study
**Plasmid VII**	pZJY4111with a fusion of *parC* and *parA*	This study
**Plasmid VIII**	pZJY4111with a 5 amino acids linker between the C and A domains of ParCA	This study
**Plasmid IX**	pZJY4111with a 8 amino acids linker between the C and A domains of ParCA	This study
**Plasmid X**	pZJY4111Δ*parC* with 42-bp deletion on the C-terminus *parA*	This study

Table S3. Primers used in this study.

Primer name	Sequence (5′–3′)*
**Primers for plasmid curing PCR detection**	
18-21-up(15505)	GTGCCCATTACTGCGTGCTAC
18-21-down(17438)	TCATTTCCGACCCTCCAACAT
APEG-up	CGCCTTGCAGATGCCCGTCC
APEG-down	TGCACGACGAGCTTGGAGGA
ori-1289-up	CGGGATCCCGAGGTCGACGGGAAGTGC
ori-4317-down	GGAATTCCGTCGACCGCAACAATCCA
**Primers for gene expression, knockout, compensation and overexpression**	
parC-F	AACATATGGGCGGGCGTCACAGGGGGGAAC
parC-R	CCTTCTGGGACACGACCGCGACAATCATTCGGCCCGCCTTCTTCGCCAGCTC
parA-F	GAGCTGGCGAAGAAGGCGGGCCGAATGATTGTCGCGGTCGTGTCCCAGAAGG
parA-R	ATGGATCCTCAACTCCCCTTCCGCTTCGTCGTG
1F	AAGAATTCGCATCGGGTGAGCGTAGAGGGT
2R	AAGGATCCGCTCATTTCCGACCCTCCAACAT
3F	AAGGATCCGATTACTCAGAGTTCGTCGAG
4R	AAGAATTCCCCACCCCAAAACGACAAGAGG
5R	GCAAGCTTCTACTTGGGGAGATACTTGTCG
6F	GGAAGCTTGGAAGTCTAGATGTCTAGATG
7R	ATCATATGTCATCGGCCCGCCTTCTTCGC
8F	CGCATATGATGTCTAGAGGTCTGGATGTC
9F	TGGCGAAGAAGGCGGGCCGATGATGGAAGTCTAGATGTCTAGATGTTTG
AF157	GTTGGCCGGTCTGCGCGTGCGGCGGTGGCGGAGGCCGGTATC
AR157	GATACCGGCCTCCGCCACCGCCGCACGCGCAGACCGGCCAAC
**Primers for parCA fusion expression**	
FC	AACATATGGGCGGGCGTCACAGGGGGGAAC
RC	CCTTCTGGGACACGACCGCGACAATCATTCGGCCCGCCTTCTTCGCCAGCTC
FA	GAGCTGGCGAAGAAGGCGGGCCGAATGATTGTCGCGGTCGTGTCCCAGAAGG
RA	ATGGATCCTCAACTCCCCTTCCGCTTCGTCGTG
Rc5	GACACGACCGCGACAATCATGCGGCGGCGGCGGCGTCGGCCCGCCTTCTTCGCCAG
Fa5	CTGGCGAAGAAGGCGGGCCGAGCGGCGGCGGCGGCGATGATTGTCGCGGTCGTGTC
Rc8	GACACGACCGCGACAATCATGCGGCGGCGGCGGCGGCGGCGGCGTCGGCCCGCCTTCT TCGCCAG
Fa8	CTGGCGAAGAAGGCGGGCCGAGCGGCGGCGGCGGCGGCGGCGGCGATGATTGTCGCGG TCGTGTC
